# Supplementary material for: Early life sleep disruption potentiates lasting sex-specific changes in behavior in genetically vulnerable Shank3 heterozygous autism model mice
Source: Mol Autism. 2022 Aug 29;13:35. doi: 10.1186/s13229-022-00514-5 (PMC9425965; doi:10.1186/s13229-022-00514-5)
Supplement: Supplementary file 1 — Additional file 1: Summary of ANOVA statistics for sleep recordings in Figure 1. [file 13229_2022_514_MOESM1_ESM.pdf]

### Juvenile male sleep data

| Light sleep amount | Mean | | SEM | ANOVA | SS | DF | MS | F (DFn | DFd) | P value | Tukey's test | Mean Diff. | 95.00% CI of diff. | Adjusted P Value | Summary |
|---|---|---|---|---|---|---|---|---|---|---|---|---|---|---|---|
| WT | 61.55 | ± | 0.963 | Genotype | 117.7 | 2 | 58.84 | F (2 | 42) = 5.516 | P=0.0075 | WT vs. HET | -0.2215 | -2.962 to 2.519 | 0.979 | ns |
| HET | 61.77 | ± | 0.656 | Residual | 448 | 42 | 10.67 | | | | WT vs. HOM | 3.633 | 0.4828 to 6.782 | 0.0205 | * |
| HOM | 57.91 | ± | 0.983 | Total | 565.7 | 44 | | | | | HET vs. HOM | 3.854 | 0.8479 to 6.860 | 0.0091 | ** |

| Dark sleep amount | Mean | | SEM | ANOVA | SS | DF | MS | F (DFn | DFd) | P value | Tukey's test | Mean Diff. | 95.00% CI of diff. | Adjusted P Value | Summary |
|---|---|---|---|---|---|---|---|---|---|---|---|---|---|---|---|
| WT | 23.23 | ± | 1.093 | Genotype | 172.2 | 2 | 86.11 | F (2 | 42) = 5.924 | P=0.0054 | WT vs. HET | 0.9953 | -2.204 to 4.195 | 0.7318 | ns |
| HET | 22.23 | ± | 0.849 | Residual | 610.5 | 42 | 14.54 | | | | WT vs. HOM | 4.997 | 1.320 to 8.674 | 0.0055 | ** |
| HOM | 18.23 | ± | 1.013 | Total | 782.7 | 44 | | | | | HET vs. HOM | 4.002 | 0.4925 to 7.511 | 0.0222 | * |

| Daily sleep amount | Mean | | SEM | ANOVA | SS | DF | MS | F (DFn | DFd) | P value | Tukey's test | Mean Diff. | 95.00% CI of diff. | Adjusted P Value | Summary |
|---|---|---|---|---|---|---|---|---|---|---|---|---|---|---|---|
| WT | 42.39 | ± | 0.506 | Genotype | 140.9 | 2 | 70.3 | F (2 | 42) = 23.48 | P=<0.0001 | WT vs. HET | 0.3869 | -1.066 to 1.840 | 0.7952 | ns |
| HET | 42 | ± | 0.434 | Residual | 126 | 42 | 3 | | | | WT vs. HOM | 4.315 | 2.645 to 5.985 | <0.0001 | **** |
| HOM | 38.07 | ± | 0.266 | Total | 266.9 | 44 | | | | | HET vs. HOM | 3.928 | 2.334 to 5.522 | <0.0001 | **** |

| Light sleep bouts | Mean | | SEM | ANOVA | SS | DF | MS | F (DFn | DFd) | P value | Tukey's test | Mean Diff. | 95.00% CI of diff. | Adjusted P Value | Summary |
|---|---|---|---|---|---|---|---|---|---|---|---|---|---|---|---|
| WT | 13.34 | ± | 0.613 | Genotype | 55153 | 2 | 27577 | F (2 | 42) = 0.8445 | P=0.4369 | WT vs. HET | n/a | | | |
| HET | 14.12 | ± | 0.784 | Residual | 1371477 | 42 | 32654 | | | | WT vs. HOM | | | | |
| HOM | 12.67 | ± | 0.912 | Total | 1426630 | 44 | | | | | HET vs. HOM | | | | |

| Dark sleep bouts | Mean | | SEM | ANOVA | SS | DF | MS | F (DFn | DFd) | P value | Tukey's test | Mean Diff. | 95.00% CI of diff. | Adjusted P Value | Summary |
|---|---|---|---|---|---|---|---|---|---|---|---|---|---|---|---|
| WT | 6.535 | ± | 0.413 | Genotype | 104032 | 2 | 52016 | F (2 | 42) = 3.177 | P=0.0519 | WT vs. HET | -78.49 | -185.9 to 28.88 | 0.19 | ns |
| HET | 7.843 | ± | 0.643 | Residual | 687635 | 42 | 16372 | | | | WT vs. HOM | 35.53 | -87.87 to 158.9 | 0.7651 | ns |
| HOM | 5.943 | ± | 0.356 | Total | 791667 | 44 | | | | | HET vs. HOM | 114 | -3.755 to 231.8 | 0.0595 | ns |

### Juvenile female sleep data

| Light sleep amount | Mean | | SEM | ANOVA | SS | DF | MS | F (DFn | DFd) | P value | Tukey's test | Mean Diff. | 95.00% CI of diff. | Adjusted P Value | Summary |
|---|---|---|---|---|---|---|---|---|---|---|---|---|---|---|---|
| WT | 59.28 | ± | 0.798 | Genotype | 111.2 | 2 | 55.59 | F (2 | 45) = 4.238 | P=0.0206 | WT vs. HET | 0.07865 | -2.741 to 2.898 | 0.9975 | ns |
| HET | 59.2 | ± | 0.825 | Residual | 590.4 | 45 | 13.12 | | | | WT vs. HOM | 3.941 | 0.3569 to 7.524 | 0.0282 | * |
| HOM | 55.34 | ± | 1.235 | Total | 701.5 | 47 | | | | | HET vs. HOM | 3.862 | 0.3647 to 7.359 | 0.0274 | * |

| Dark sleep amount | Mean | | SEM | ANOVA | SS | DF | MS | F (DFn | DFd) | P value | Tukey's test | Mean Diff. | 95.00% CI of diff. | Adjusted P Value | Summary |
|---|---|---|---|---|---|---|---|---|---|---|---|---|---|---|---|
| WT | 22.16 | ± | 0.945 | Genotype | 95.98 | 2 | 47.99 | F (2 | 45) = 4.341 | P=0.0189 | WT vs. HET | 0.221 | -2.367 to 2.809 | 0.9767 | ns |
| HET | 21.94 | ± | 0.627 | Residual | 497.5 | 45 | 11.05 | | | | WT vs. HOM | 3.733 | 0.4432 to 7.023 | 0.0228 | * |
| HOM | 18.42 | ± | 0.906 | Total | 593.5 | 47 | | | | | HET vs. HOM | 3.512 | 0.3015 to 6.722 | 0.0292 | * |

| Daily sleep amount | Mean | | SEM | ANOVA | SS | DF | MS | F (DFn | DFd) | P value | Tukey's test | Mean Diff. | 95.00% CI of diff. | Adjusted P Value | Summary |
|---|---|---|---|---|---|---|---|---|---|---|---|---|---|---|---|
| WT | 40.72 | ± | 0.377 | Genotype | 103.4 | 2 | 51.69 | F (2 | 45) = 10.25 | P=0.0002 | WT vs. HET | 0.1498 | -1.599 to 1.898 | 0.9765 | ns |
| HET | 40.57 | ± | 0.535 | Residual | 227 | 45 | 5.044 | | | | WT vs. HOM | 3.837 | 1.615 to 6.059 | 0.0004 | *** |
| HOM | 36.88 | ± | 0.939 | Total | 330.4 | 47 | | | | | HET vs. HOM | 3.687 | 1.518 to 5.856 | 0.0005 | *** |

| Light sleep bouts | Mean | | SEM | ANOVA | SS | DF | MS | F (DFn | DFd) | P value | Tukey's test | Mean Diff. | 95.00% CI of diff. | Adjusted P Value | Summary |
|---|---|---|---|---|---|---|---|---|---|---|---|---|---|---|---|
| WT | 12.25 | ± | 0.662 | Genotype | 250397 | 2 | 125199 | F (2 | 45) = 4.512 | P=0.0164 | WT vs. HET | -59.71 | -189.4 to 69.97 | 0.5094 | ns |
| HET | 13.24 | ± | 0.639 | Residual | 1248782 | 45 | 27751 | | | | WT vs. HOM | 139.7 | -25.17 to 304.5 | 0.1113 | ns |
| HOM | 9.921 | ± | 0.756 | Total | 1499179 | 47 | | | | | HET vs. HOM | 199.4 | 38.51 to 360.2 | 0.0119 | * |

| Dark sleep bouts | Mean | | SEM | ANOVA | SS | DF | MS | F (DFn | DFd) | P value | Tukey's test | Mean Diff. | 95.00% CI of diff. | Adjusted P Value | Summary |
|---|---|---|---|---|---|---|---|---|---|---|---|---|---|---|---|
| WT | 6.732 | ± | 0.602 | Genotype | 69545 | 2 | 34772 | F (2 | 45) = 1.528 | P=0.2281 | WT vs. HET | n/a | | | |
| HET | 7.654 | ± | 0.596 | Residual | 1024340 | 45 | 22763 | | | | WT vs. HOM | | | | |
| HOM | 5.998 | ± | 0.581 | Total | 1093885 | 47 | | | | | HET vs. HOM | | | | |

### Adolescent male sleep data

| Light sleep amount | Mean | | SEM | ANOVA | SS | DF | MS | F (DFn | DFd) | P value | Tukey's test | Mean Diff. | 95.00% CI of diff. | Adjusted P Value | Summary |
|---|---|---|---|---|---|---|---|---|---|---|---|---|---|---|---|
| WT | 61.87 | ± | 0.962 | Genotype | 74.29 | 2 | 37.15 | F (2 | 39) = 2.410 | P=0.1031 | WT vs. HET | n/a | | | |
| HET | 59.48 | ± | 0.962 | Residual | 601.1 | 39 | 15.41 | | | | WT vs. HOM | | | | |
| HOM | 58.53 | ± | 1.219 | Total | 675.4 | 41 | | | | | HET vs. HOM | | | | |

| Dark sleep amount | Mean | | SEM | ANOVA | SS | DF | MS | F (DFn | DFd) | P value | Tukey's test | Mean Diff. | 95.00% CI of diff. | Adjusted P Value | Summary |
|---|---|---|---|---|---|---|---|---|---|---|---|---|---|---|---|
| WT | 24.45 | ± | 0.92 | Genotype | 237.7 | 2 | 118.9 | F (2 | 39) = 4.145 | P=0.0233 | WT vs. HET | -2.909 | -7.336 to 1.518 | 0.2573 | ns |
| HET | 27.36 | ± | 1.373 | Residual | 1118 | 39 | 28.68 | | | | WT vs. HOM | 3.727 | -2.185 to 9.640 | 0.2856 | ns |
| HOM | 20.72 | ± | 2.537 | Total | 1356 | 41 | | | | | HET vs. HOM | 6.636 | 0.8676 to 12.40 | 0.021 | * |

| Daily sleep amount | Mean | | SEM | ANOVA | SS | DF | MS | F (DFn | DFd) | P value | Tukey's test | Mean Diff. | 95.00% CI of diff. | Adjusted P Value | Summary |
|---|---|---|---|---|---|---|---|---|---|---|---|---|---|---|---|
| WT | 43.16 | ± | 0.433 | Genotype | 79.44 | 2 | 39.72 | F (2 | 39) = 8.777 | P=0.0007 | WT vs. HET | -0.2568 | -2.015 to 1.502 | 0.9327 | ns |
| HET | 43.42 | ± | 0.485 | Residual | 176.5 | 39 | 4.525 | | | | WT vs. HOM | 3.538 | 1.189 to 5.886 | 0.0021 | ** |
| HOM | 39.62 | ± | 1.103 | Total | 255.9 | 41 | | | | | HET vs. HOM | 3.794 | 1.503 to 6.086 | 0.0007 | *** |

| Light sleep bouts | Mean | | SEM | ANOVA | SS | DF | MS | F (DFn | DFd) | P value | Tukey's test | Mean Diff. | 95.00% CI of diff. | Adjusted P Value | Summary |
|---|---|---|---|---|---|---|---|---|---|---|---|---|---|---|---|
| WT | 12.25 | ± | 0.772 | Genotype | 142057 | 2 | 71029 | F (2 | 39) = 3.277 | P=0.0484 | WT vs. HET | 88.44 | -33.27 to 210.2 | 0.1929 | ns |
| HET | 10.78 | ± | 0.49 | Residual | 845436 | 39 | 21678 | | | | WT vs. HOM | 160.7 | -1.819 to 323.3 | 0.0532 | ns |
| HOM | 9.574 | ± | 0.48 | Total | 987493 | 41 | | | | | HET vs. HOM | 72.3 | -86.30 to 230.9 | 0.5134 | ns |

| Dark sleep bouts | Mean | | SEM | ANOVA | SS | DF | MS | F (DFn | DFd) | P value | Tukey's test | Mean Diff. | 95.00% CI of diff. | Adjusted P Value | Summary |
|---|---|---|---|---|---|---|---|---|---|---|---|---|---|---|---|
| WT | 6.565 | ± | 0.554 | Genotype | 79073 | 2 | 39537 | F (2 | 39) = 4.481 | P=0.0177 | WT vs. HET | 65.53 | -12.12 to 143.2 | 0.1125 | ns |
| HET | 5.473 | ± | 0.225 | Residual | 344105 | 39 | 8823 | | | | WT vs. HOM | 120.2 | 16.50 to 223.9 | 0.0199 | * |
| HOM | 4.562 | ± | 0.326 | Total | 423178 | 41 | | | | | HET vs. HOM | 54.68 | -46.51 to 155.9 | 0.3947 | ns |

### Adolescent female sleep data

| Light sleep amount | Mean | | SEM | ANOVA | SS | DF | MS | F (DFn | DFd) | P value | Tukey's test | Mean Diff. | 95.00% CI of diff. | Adjusted P Value | Summary |
|---|---|---|---|---|---|---|---|---|---|---|---|---|---|---|---|
| WT | 58.8 | ± | 1.004 | Genotype | 107.6 | 2 | 53.82 | F (2 | 30) = 3.816 | P=0.0334 | WT vs. HET | 3.016 | -0.6907 to 6.722 | 0.1282 | ns |
| HET | 55.79 | ± | 1.354 | Residual | 423.1 | 30 | 14.1 | | | | WT vs. HOM | 4.345 | 0.1849 to 8.506 | 0.0392 | * |
| HOM | 54.46 | ± | 0.651 | Total | 530.8 | 32 | | | | | HET vs. HOM | 1.33 | -2.896 to 5.555 | 0.7206 | ns |

| Dark sleep amount | Mean | | SEM | ANOVA | SS | DF | MS | F (DFn | DFd) | P value | Tukey's test | Mean Diff. | 95.00% CI of diff. | Adjusted P Value | Summary |
|---|---|---|---|---|---|---|---|---|---|---|---|---|---|---|---|
| WT | 22.46 | ± | 0.927 | Genotype | 103.7 | 2 | 51.85 | F (2 | 30) = 2.991 | P=0.0654 | WT vs. HET | -2.06 | -6.169 to 2.049 | 0.4419 | ns |
| HET | 24.52 | ± | 1.434 | Residual | 520 | 30 | 17.33 | | | | WT vs. HOM | 2.581 | -2.031 to 7.193 | 0.3641 | ns |
| HOM | 19.88 | ± | 1.43 | Total | 623.7 | 32 | | | | | HET vs. HOM | 4.641 | -0.04413 to 9.326 | 0.0526 | ns |

| Daily sleep amount | Mean | | SEM | ANOVA | SS | DF | MS | F (DFn | DFd) | P value | Tukey's test | Mean Diff. | 95.00% CI of diff. | Adjusted P Value | Summary |
|---|---|---|---|---|---|---|---|---|---|---|---|---|---|---|---|
| WT | 40.63 | ± | 0.527 | Genotype | 64.8 | 2 | 32.4 | F (2 | 30) = 5.564 | P=0.0088 | WT vs. HET | 0.478 | -1.904 to 2.859 | 0.8743 | ns |
| HET | 40.15 | ± | 0.86 | Residual | 174.7 | 30 | 5.823 | | | | WT vs. HOM | 3.463 | 0.7899 to 6.136 | 0.009 | ** |
| HOM | 37.17 | ± | 0.777 | Total | 239.5 | 32 | | | | | HET vs. HOM | 2.985 | 0.2698 to 5.701 | 0.0288 | * |

| Light sleep bouts | Mean | | SEM | ANOVA | SS | DF | MS | F (DFn | DFd) | P value | Tukey's test | Mean Diff. | 95.00% CI of diff. | Adjusted P Value | Summary |
|---|---|---|---|---|---|---|---|---|---|---|---|---|---|---|---|
| WT | 11.83 | ± | 0.628 | Genotype | 183787 | 2 | 91894 | F (2 | 30) = 5.810 | P=0.0074 | WT vs. HET | 61.59 | -62.53 to 185.7 | 0.449 | ns |
| HET | 10.81 | ± | 0.664 | Residual | 474502 | 30 | 15817 | | | | WT vs. HOM | 192.1 | 52.78 to 331.4 | 0.0053 | ** |
| HOM | 8.632 | ± | 0.463 | Total | 658289 | 32 | | | | | HET vs. HOM | 130.5 | -11.00 to 272.0 | 0.0752 | ns |

| Dark sleep bouts | Mean | | SEM | ANOVA | SS | DF | MS | F (DFn | DFd) | P value | Tukey's test | Mean Diff. | 95.00% CI of diff. | Adjusted P Value | Summary |
|---|---|---|---|---|---|---|---|---|---|---|---|---|---|---|---|
| WT | 7.379 | ± | 0.729 | Genotype | 119325 | 2 | 59663 | F (2 | 30) = 4.071 | P=0.0273 | WT vs. HET | 44.65 | -74.82 to 164.1 | 0.6312 | ns |
| HET | 6.635 | ± | 0.394 | Residual | 439672 | 30 | 14656 | | | | WT vs. HOM | 154.2 | 20.12 to 288.3 | 0.0215 | * |
| HOM | 4.809 | ± | 0.578 | Total | 558997 | 32 | | | | | HET vs. HOM | 109.6 | -26.64 to 245.8 | 0.1338 | ns |
