# Supplementary material for: Early life sleep disruption potentiates lasting sex-specific changes in behavior in genetically vulnerable Shank3 heterozygous autism model mice
Source: Mol Autism. 2022 Aug 29;13:35. doi: 10.1186/s13229-022-00514-5 (PMC9425965; doi:10.1186/s13229-022-00514-5)
Supplement: Supplementary file 2 — Additional file 2: Summary of statistics for behavior tests in Figures 3 and 5. [file 13229_2022_514_MOESM2_ESM.pdf]

Elevated plus maze

Percent time in open arms	Means ± SEM		Unpaired t-test with Holm-Šídák correction		
	**WT**	**HET**	**t ratio**	**df**	**p value**
**Control males**	25.91 ± 4.78	26.31 ± 3.12	0.06472	17	0.964152
**ELSD males**	24.98 ± 4.98	23.19 ± 5.37	0.2429	19	0.964152
**PASD males**	33.40 ± 6.84	38.49 ± 2.86	0.6866	14	0.877635
**Control females**	17.69 ± 1.94	22.49 ± 6.21	0.8093	16	0.43021
**** ELSD females**	23.83 ± 2.75	36.56 ± 2.13	3.51	16	0.008685
**# PASD females**	32.75 ± 4.40	46.10 ± 4.40	2.148	22	0.084182

Percent open arm entrances	Means ± SEM		Unpaired t-test with Holm-Šídák correction		
	**WT**	**HET**	**t ratio**	**df**	**p value**
**Control males**	36.04 ± 3.47	34.51 ± 2.28	0.3363	17	0.766385
**ELSD males**	32.95 ± 2.40	30.21 ± 2.94	0.7133	19	0.766385
**PASD males**	37.13 ± 5.02	41.75 ± 1.13	0.8985	14	0.766385
**Control females**	31.08 ± 3.31	31.61 ± 1.77	0.1317	16	0.896834
**# ELSD females**	34.55 ± 2.41	43.38 ± 2.41	2.557	16	0.061947
**PASD females**	37.20 ± 2.93	42.44 ± 2.87	1.28	22	0.38217

Open field test

Total distance traveled (cm)	Means ± SEM		Unpaired t-test with Holm-Šídák correction		
	**WT**	**HET**	**t ratio**	**df**	**p value**
**Control males**	6117.64 ± 519.83	6812.57 ± 407.81	0.9489	16	0.356784
**** ELSD males**	7675.30 ± 687.29	5211.27 ± 478.36	2.988	19	0.022514
**PASD males**	7402.25 ± 584.49	6329.25 ± 167.60	1.765	14	0.188948
**Control females**	5804.90 ± 505.58	6679.00 ± 458.05	1.25	16	0.405805
**ELSD females**	6831.00 ± 846.82	6798.75 ± 530.14	0.03132	15	0.975429
**PASD females**	8946.58 ± 845.93	6863.42 ± 480.23	2.142	22	0.125066

Prepulse inhibition

Percent startle inhibition	*Means ± SEM*		*2-way ANOVA*				*Šídák's multiple comparisons test*	
**Control males**	**WT**	**HET**	**Factor**	**% variation**	**F(DFn, DFd)**	**p value**		
Startle tone (dB) 74	12.27 ± 11.57	13.05 ± 6.73	dB x gtype	0.2831	F (4, 68) = 0.2549	0.9057	-	-
78	55.21 ± 3.30	48.98 ± 4.62	dB trial	65.31	F (1.569, 26.67) = 58.81	<0.0001		
82	65.48 ± 3.92	66.49 ± 3.60	genotype	0.2335	F (1, 17) = 0.3079	0.5862		
86	76.72 ± 3.58	72.65 ± 3.66	subject	12.9	F (17, 68) = 2.733	0.0018		
90	81.45 ± 2.93	75.70 ± 3.05						
**ELSD males**	**WT**	**HET**	**Factor**	**% variation**	**F(DFn, DFd)**	**p value**	**dB**	**Adjusted p value**
74	28.69 ± 5.09	18.16 ± 9.87	dB x gtype	0.2965	F (4, 76) = 0.4587	0.7658	74	0.8913
78	65.11 ± 3.87	46.78 ± 8.62	dB trial	51.84	F (2.234, 42.44) = 80.18	<0.0001	78	0.3159
82	78.92 ± 2.75	59.67 ± 6.59	genotype	8.412	F (1, 19) = 5.847	0.0258	82	0.0869
*86	84.00 ± 2.70	67.71 ± 3.89	subject	27.34	F (19, 76) = 8.902	<0.0001	86	0.015
**90	86.19 ± 1.26	69.75 ± 3.98					90	0.0099
**PASD males**	**WT**	**HET**	**Factor**	**% variation**	**F(DFn, DFd)**	**p value**		
74	22.40 ± 7.47	25.41 ± 10.32	dB x gtype	2.183	F (4, 56) = 2.374	0.063	-	-
78	42.78 ± 8.39	66.73 ± 6.99	dB trial	47.33	F (1.909, 26.72) = 51.48	<0.0001		
82	63.65 ± 8.94	73.04 ± 5.32	genotype	2.788	F (1, 14) = 1.121	0.3077		
86	67.36 ± 7.45	77.08 ± 5.23	subject	34.83	F (14, 56) = 10.82	<0.0001		
90	76.48 ± 5.87	76.74 ± 4.15						
**Control females**	**WT**	**HET**	**Factor**	**% variation**	**F(DFn, DFd)**	**p value**		
74	25.86 ± 7.56	28.38 ± 5.30	dB x gtype	1.26	F (4, 64) = 1.107	0.3609	-	-
78	62.79 ± 3.91	52.80 ± 6.68	dB trial	53.81	F (2.093, 33.48) = 47.29	<0.0001		
82	68.70 ± 3.54	57.49 ± 5.99	genotype	1.74	F (1, 16) = 1.209	0.2879		
86	71.20 ± 3.64	67.46 ± 3.28	subject	23.03	F (16, 64) = 5.060	<0.0001		
90	74.65 ± 3.01	68.21 ± 4.64						
**ELSD females**	**WT**	**HET**	**Factor**	**% variation**	**F(DFn, DFd)**	**p value**		
74	30.28 ± 10.84	11.98 ± 9.72	dB x gtype	1.577	F (4, 64) = 1.896	0.122	-	-
78	59.81 ± 6.21	60.11 ± 5.13	dB trial	51.54	F (1.553, 24.85) = 61.94	<0.0001		
82	66.80 ± 6.62	67.88 ± 5.80	genotype	0.7215	F (1, 16) = 0.3407	0.5676		
86	78.05 ± 3.90	72.46 ± 6.42	subject	33.88	F (16, 64) = 10.18	<0.0001		
90	77.42 ± 4.73	75.84 ± 5.05						
**PASD females**	**WT**	**HET**	**Factor**	**% variation**	**F(DFn, DFd)**	**p value**	**dB**	**Adjusted p value**
*74	41.56 ± 8.21	11.48 ± 6.32	dB x gtype	3.45	F (4, 88) = 4.895	0.0013	74	0.0423
78	64.63 ± 3.36	59.28 ± 3.20	dB trial	59.59	F (1.363, 29.98) = 84.56	<0.0001	78	0.7797
82	73.93 ± 3.15	65.39 ± 3.47	genotype	5.679	F (1, 22) = 7.916	0.0101	82	0.3489
86	80.34 ± 1.70	72.93 ± 3.14	subject	15.78	F (22, 88) = 4.072	<0.0001	86	0.2393
90	83.07 ± 1.88	75.45 ± 2.69					90	0.1472

Sociability

Seconds in proximity Means ± SEM — Paired t-test with Holm-Šídák correction

		non-social			stranger 1				t	df	p value
**Control males**		**non-social**			**stranger 1**				**t**	**df**	**p value**
	WT	75.17	±	8.25	145.26	±	18.84	WT	3.161	10	0.020179
	HET	81.18	±	8.68	161.48	±	24.76	HET	2.544	7	0.038447
**ELSD males**		**non-social**			**stranger 1**				**t**	**df**	**p value**
	WT	78.54	±	7.35	157.93	±	12.21	WT	5.723	9	0.000571
	HET	92.20	±	5.54	117.01	±	11.81	HET	1.674	10	0.125092
**PASD males**		**non-social**			**stranger 1**				**t**	**df**	**p value**
	WT	71.30	±	5.61	158.71	±	11.65	WT	7.071	7	0.000201
	HET	68.65	±	6.41	144.16	±	7.28	HET	7.881	7	0.000201
**Control females**		**non-social**			**stranger 1**				**t**	**df**	**p value**
	WT	72.93	±	7.83	152.36	±	15.69	WT	4.812	8	0.001334
	HET	75.69	±	7.29	147.53	±	6.10	HET	9.265	7	0.000071
**ELSD females**		**non-social**			**stranger 1**				**t**	**df**	**p value**
	WT	80.74	±	6.40	168.75	±	19.48	WT	3.823	9	0.00812
	HET	99.78	±	15.06	182.89	±	17.83	HET	2.791	7	0.026867
**PASD females**		**non-social**			**stranger 1**				**t**	**df**	**p value**
	WT	73.63	±	7.00	111.64	±	10.06	WT	2.807	11	0.017072
	HET	76.98	±	6.84	132.80	±	9.93	HET	4.263	11	0.002672

Social novelty preference

Seconds in proximity Means ± SEM — Paired t-test with Holm-Šídák correction

**Control males**		**stranger 1**			**stranger 2**				**t**	**df**	**p value**
	WT	68.88	±	10.20	125.45	±	9.88	WT	4.588	10	0.001995
	HET	64.55	±	10.04	133.73	±	17.43	HET	2.663	7	0.032307
**ELSD males**		**stranger 1**			**stranger 2**				**t**	**df**	**p value**
	WT	71.78	±	10.06	137.16	±	14.21	WT	3.581	9	0.005919
	HET	56.25	±	8.98	167.87	±	16.85	HET	5.955	10	0.000281
**PASD males**		**stranger 1**			**stranger 2**				**t**	**df**	**p value**
	WT	62.48	±	7.41	120.98	±	14.94	WT	3.955	7	0.010959
	HET	68.65	±	10.37	95.31	±	5.22	HET	2.003	7	0.085182
**Control females**		**stranger 1**			**stranger 2**				**t**	**df**	**p value**
	WT	64.03	±	10.73	123.38	±	10.09	WT	3.93	8	0.008699
	HET	98.38	±	16.17	121.80	±	13.61	HET	1.057	7	0.325736
**ELSD females**		**stranger 1**			**stranger 2**				**t**	**df**	**p value**
	WT	64.92	±	7.41	118.66	±	8.99	WT	5.147	9	0.001211
	HET	75.29	±	13.98	111.31	±	9.76	HET	1.677	7	0.137533
**PASD females**		**stranger 1**			**stranger 2**				**t**	**df**	**p value**
	WT	69.54	±	10.67	90.07	±	12.13	WT	1.092	11	0.298247
	HET	82.92	±	13.20	117.41	±	18.03	HET	1.781	11	0.194496
