# Supplementary figures and images for: Early life sleep disruption potentiates lasting sex-specific changes in behavior in genetically vulnerable Shank3 heterozygous autism model mice
Source: Mol Autism. 2022 Aug 29;13:35. doi: 10.1186/s13229-022-00514-5 (PMC9425965; doi:10.1186/s13229-022-00514-5)

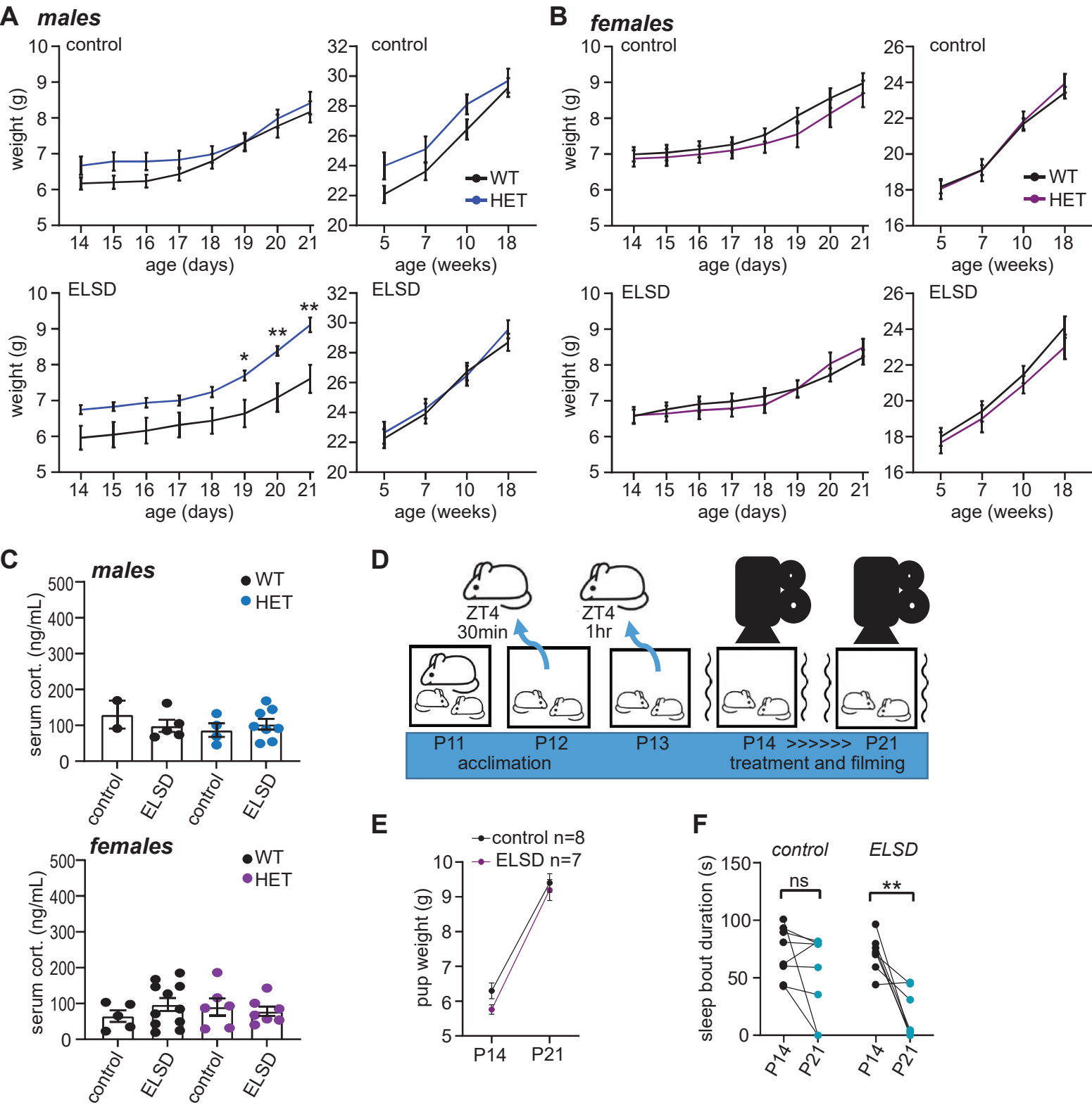

Supplement: Supplementary file 3 — Additional file 3: Weight gain, corticosterone levels, and in-treatment sleep bouts in control and ELSD cohorts. (A-B) Control and ELSD cohorts were weighed daily from P14-P21 to ensure that pups were receiving sufficient maternal care during ELSD treatment, and at multiple time points during the remainder of the experiment. (A) weight of male control and ELSD cohorts. Shank3WT/ΔC heterozygotes (HET) exposed to ELSD gain weight faster than WT littermates (2-way ANOVA: main effect of genotype: F(1,19) = 6.766, p = 0.175); however, this difference was normalized by 5 weeks of age. N=8-11 per treatment/genotype. *P<0.05, **P<0.01 (2-way ANOVA). (B) Weight of female control and ELSD cohorts. No differences in weight between treatments or genotypes was observed (2-way ANOVA). N=8-12 per treatment/genotype. (C) Serum corticosterone (cort.) was measured using ELISA from separate cohorts of control and ELSD treated pups at P21. No differences in cort. levels between treatments or genotypes within each sex were observed (1-way ANOVA). (D-F) two ELSD and two CON litters were filmed to confirm sleep disruption; (D) experimental design. Pups were acclimated to dam removal at ZT4 prior to live-video recording on P14 and P21. (E) ELSD did not affect weight gain. (F) Seven days of ELSD treatment significantly reduced sleep bout duration by postnatal day 21 **P<0.01 (paired t-test, control: t(7) = 1.484; ELSD: t(6) = 3.923). N=7-8 per treatment. [file 13229_2022_514_MOESM3_ESM.pdf]

Additional File 4

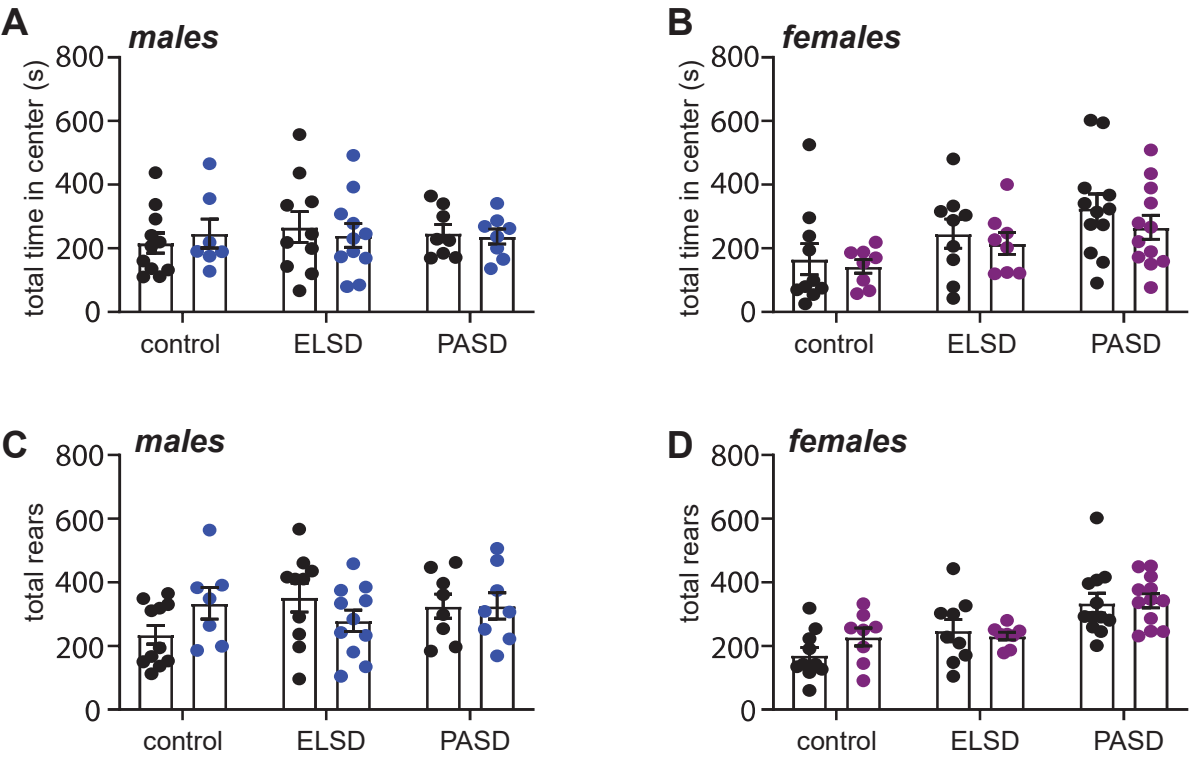

Supplement: Supplementary file 4 — Additional file 4: No changes in anxiety-like measures in the open field test. (A-B) no differences observed in total time spent in the center of the open field from control, ELSD, and PASD treated WT and Shank3WT/ΔC heterozygous (HET) males (A) and females (B). (C-D) no differences observed in number of rears during the open field test from control, ELSD, and PASD treated males (C) and females (D). (unpaired t-tests with Holm-Šídák correction). N=8-12 per treatment/sex/genotype. [file 13229_2022_514_MOESM4_ESM.pdf]
